# Treatment of Neovascular Age-Related Macular Degeneration with Anti-VEGF Agents: Predictive Factors of Long-Term Visual Outcomes

**DOI:** 10.1155/2017/4263017

**Published:** 2017-06-01

**Authors:** Ana Catarina Pedrosa, Tiago Sousa, João Pinheiro-Costa, João Beato, Manuel S. Falcão, Fernando Falcão-Reis, Angela Carneiro

**Affiliations:** ^1^Department of Ophthalmology, Centro Hospitalar São João, Porto, Portugal; ^2^Department of Sense Organs, Faculty of Medicine of the University of Porto, Porto, Portugal; ^3^Department of Anatomy, Faculty of Medicine of the University of Porto, Porto, Portugal

## Abstract

**Purpose:**

To evaluate the predictive factors of long-term visual outcomes in neovascular age-related macular degeneration (nAMD) treated with antivascular endothelial growth factor (anti-VEGF) agents.

**Methods:**

Unicentric retrospective review of patients with nAMD treated with anti-VEGF agents. Visual outcomes, 12 and 60 months after diagnosis, were evaluated. In an attempt to identify predictive factors of visual outcomes, multiple variables (demographic and epidemiological characteristics, angiographic and tomographic features) were analyzed, at baseline and during follow-up.

**Results:**

One hundred and seventeen patients were included. In multivariate analysis, baseline best-corrected visual acuity was associated with all visual endpoints at 12 and 60 months. Additionally, age, gender, number of injections, and development of subretinal fibrosis during follow-up were also significant predictors of visual outcomes at 60 months.

**Conclusions:**

Several factors can be useful in clinical practice as predictors of visual outcomes in response to anti-VEGF treatment of nAMD.

## 1. Introduction

In developed countries, age-related macular degeneration (AMD) is the leading cause of visual loss in individuals over 55 years of age [1]. While representing only 10–20% of AMD cases, the neovascular subtype (nAMD) accounts for 80–90% of severe vision loss in AMD [1].

The visual prognosis of nAMD was dramatically improved by the introduction of antivascular endothelial growth factor (anti-VEGF) agents into clinical practice. The natural history of nAMD is characterized by relatively rapid and inexorably progressive visual loss, with almost 50% of patients losing at least three lines of vision over two years [2]. In contrast, about 80% of patients under monthly ranibizumab treatment avoid visual loss (of >0 early treatment diabetic retinopathy study (ETDRS) letters) over the same time period [3].

Although the overall effectiveness of anti-VEGF therapy is unquestionable, however, there is individual variability in clinical response. While, on average, visual outcomes were excellent in the ANCHOR [3] and MARINA [2] trials, with a mean increase of 11 and 7 ETDRS letters, respectively, after two years of monthly ranibizumab treatment, 10% of patients still lost at least three lines of vision despite adequate treatment [2, 3]. Additionally, those who improved did not all benefit in the same manner; indeed, 33–41% were particularly sensitive to treatment, showing an impressive gain of three or more lines of vision at two years [2, 3].

Being able to predict the individual response to anti-VEGF therapy could be important for several reasons: (1) it might allow ophthalmologists and their patients to adjust their expectations for visual outcomes; (2) it might help optimize treatment, by enabling, for example, the identification of patients who require more frequent anti-VEGF injections or complementary treatments; (3) it might further expand our knowledge on the pathogenesis of nAMD, leading to the development of alternative or complementary treatment strategies.

In our study, we aimed to identify predictive factors of visual outcomes, at one and five years of follow-up, in nAMD patients treated with anti-VEGF agents.

## 2. Methods

### 2.1. Clinical Setting

This was a retrospective study performed at the Department of Ophthalmology of Centro Hospitalar de São João, Porto, Portugal. The study complies with the ethical principles set by the Declaration of Helsinki, and approval for it was obtained from the Ethics Committee of the hospital and of the associated Faculty of Medicine of the University of Porto.

### 2.2. Selection of Participants

We included all patients with nAMD, who began anti-VEGF treatment at our center before October 2009 and who had at least five years of continuous follow-up. Anti-VEGF treatment could include bevacizumab, ranibizumab, and/or aflibercept. Patients were excluded whenever best-corrected visual acuity (BCVA) at diagnosis was inferior to 10 ETDRS letters or whenever the study eye had received photodynamic therapy prior to or concomitantly with anti-VEGF injections. Only one eye of each patient was selected: if both eyes fulfilled the selection criteria, we arbitrarily selected the right one.

In all cases, the diagnosis of nAMD was based on funduscopic examination, optical coherence tomography (OCT), and fluorescein angiography, which were routinely performed at baseline.

### 2.3. Anti-VEGF Agents and Treatment Regimens

At our center, anti-VEGF therapy changed over time, as new scientific evidence emerged and board decisions were made based on that evidence. Anti-VEGF therapy of nAMD began in 2006 with the off-label use of bevacizumab. Later, in 2008, ranibizumab became commercially available and replaced bevacizumab. However, in 2011, after the CATT study demonstrated that they had equivalent effects, bevacizumab became once again the drug of choice and ranibizumab became available only for nonresponders. Finally, in 2013, aflibercept replaced ranibizumab as rescue therapy.

In 2006, when anti-VEGF therapy began, all patients were treated as-needed with monthly monitoring, but this strategy proved to be very demanding in terms of time and human resources. Therefore, as the number of patients under treatment increased, we gradually changed to a regimen similar to “treat-and-extend,” where the interval between injections is progressively lengthened by one week on each medical visit, as long as there are no signs of neovascular activity. When a 12-week interval is reached, treatment may be suspended. Due to practical constraints imposed by the rapidly growing number of patients, when a medical visit cannot be scheduled on the same week as the following intravitreal injection, patients may have two or three consecutive injections with fixed intervals before returning for their appointment. However, they are advised to immediately contact their Ophthalmologist if they develop any alarm symptoms, such as decreasing visual acuity, metamorphopsia, or new scotomas.

In both treatment regimens, neovascular activity was assessed based on the presence of new macular exudates or hemorrhages on funduscopic examination, intraretinal or subretinal fluid on OCT, and/or active leakage on fluorescein angiography (which was performed during follow-up at the discretion of the clinician).

At our center, the medical visit and the intravitreal injection may be performed on different days. Treatment takes place in the operating room, under topical anesthesia. The dose of anti-VEGF agents administered is 0.5 mg/ 50 *μ*L for ranibizumab, 1.25 mg/ 50 *μ*L for bevacizumab, and 2.0 mg/50 *μ*L for aflibercept.

OCT scans were obtained with the Stratus device (Carl Zeiss Meditec AG, Jena, Germany) until 2009 and with the Spectralis HRA + OCT device (Heidelberg Engineering GmbH, Heidelberg, Germany) thereafter.

### 2.4. Collection of Data

Relevant data was collected retrospectively from patients' records.

In our search for predictors of visual outcomes, we considered, at baseline, the following factors: age, gender, BCVA, treatment delay (defined as the interval between the onset of symptoms and the first anti-VEGF injection), type of choroidal neovascularization (CNV) lesion (occult, minimally classic, predominantly classic, or retinal angiomatous proliferation), central foveal thickness (CFT), presence of subretinal fibrosis, and presence of subfoveal hemorrhage. During follow-up, we also analyzed the frequency of medical visits and of missed medical visits, the frequency of intravitreal injections and of missed intravitreal injections, the frequency of treatment interruptions, and the development of retinal atrophy, subretinal fibrosis, massive hemorrhage, or retinal pigment epithelium (RPE) tear. We considered that treatment was interrupted when (a) in the pro re nata (PRN) regimen, at a certain visit, the patient did not present criteria for retreatment, or (b) in the treat-and-extend regimen, after progressively lengthening the interval between injections to 12 weeks, the clinician decided to stop treatment and monitor the patient, or (c) the patient missed a planned injection.

Visual outcomes were analyzed at one and five years of follow-up, and they included final BCVA score, final BCVA ≥ 65 ETDRS letters, BVCA variation, and BCVA variation > 0 ETDRS letters.

### 2.5. Statistical Analysis

Beforehand, we conducted a univariate analysis using the appropriate statistical test regarding the four outcomes, in order to evaluate the eligibility of inclusion for each variable in the final multivariate analysis, using a *p*  value < 0.20 as criteria of inclusion. The final multivariate models were then conducted applying a backward selection method, retaining only those variables with *p*  value < 0.05, using multiple linear regression (regarding the “BCVA” and “BCVA variation” outcomes) and logistic regression (regarding the “BCVA ≥ 65 letters” and “BCVA variation > 0 letters” outcomes). Statistical analysis was performed using the IBM® SPSS® Statistics statistical software for Windows, Version 23.0 (Armonk, NY: IBM Corp.).

## 3. Results

A total of 117 patients met the selection criteria, 58.1% of whom were female. Age at diagnosis was, on average, 76 and varied between 54 and 89 years. Mean baseline BCVA in the study eye was 47 ± 19.6 ETDRS letters. Angiographically, CNV lesions were classified as occult in 47.0%, minimally classic in 29.9%, predominantly classic in 13.7%, and retinal angiomatous proliferation in 6.8% of cases (in the remaining 2.6%, the CNV lesion was not classified in the patient's records). At baseline, mean CFT was 311.37 ± 140.85 *μ*m, subfoveal hemorrhage was present in 8.5% of patients, and subretinal fibrosis was present in 7.7% of patients (Table 1).

On average, each patient attended 9.3 medical visits during the first year, and this number progressively decreased to 6.1 in the fifth year. The mean number of missed medical visits during the five-year follow-up period was 1.6, most of which were due to other diseases (59.0%). Seventy-eight (66.7%) patients missed at least one medical visit, while 49 (41.9%) missed more than one. On average, each patient received 6.0 injections during the first year; the annual number decreased to around four injections thereafter. Twenty-five (21.4%) patients missed at least one injection, while only two (1.7%) missed more than one. On average, each patient had 5.3 treatment interruptions over five years, the majority of which (90.8%) were due to medical recommendation. During follow-up, 83 (70.9%) patients developed atrophy in the study eye (62.7% of them during treatment), nine (7.7%) developed RPE tear (88.9% of them during treatment), and seventy-five (68.5%) developed subretinal fibrosis (81.3% of them during treatment). Thirty-eight (32.5%) patients had at least one sudden massive hemorrhage, while nine (7.7%) had more than one; the frequency of these events was similar in the treatment and suspension periods (51.1% and 48.9%, resp.) (Table 2).

### 3.1. Predictors for Outcomes at 12 Months

**Figure 1:**
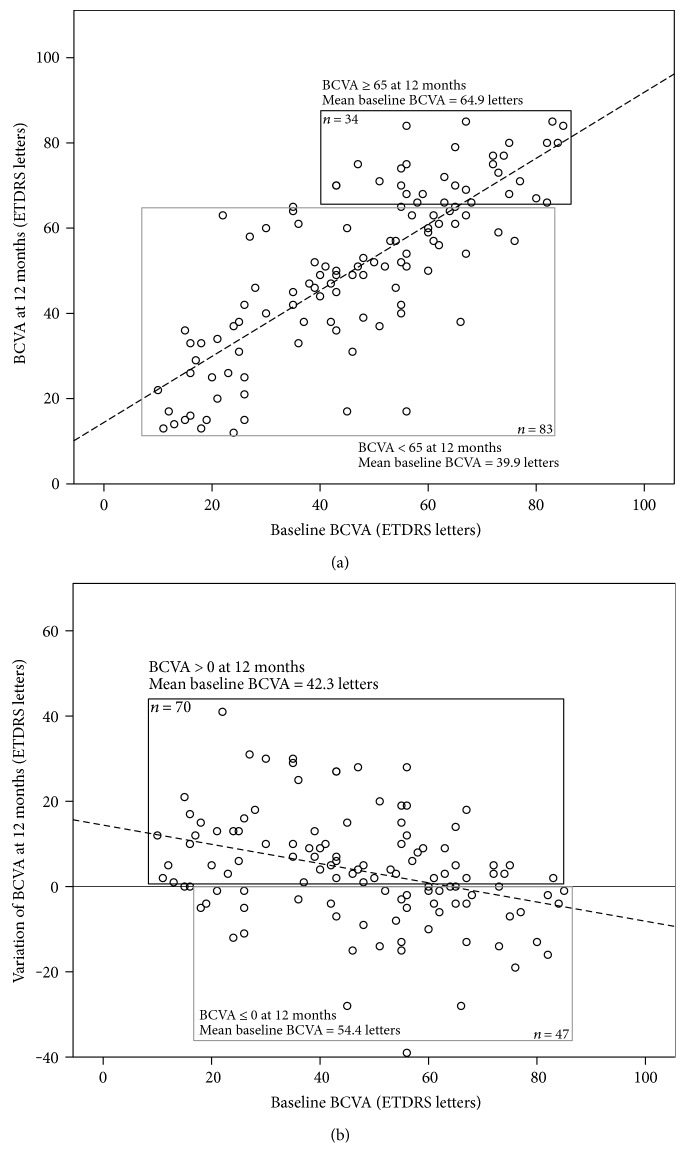
Predictive variables at 12 months. (a) Better BCVA at baseline predicted higher final BCVA score and greater likelihood of achieving 65 ETDRS letters or better, at 12 months. Dashed line = fitted simple linear regression line. (b) Worse BCVA at baseline predicted higher BCVA variation and greater likelihood of achieving a variation greater than 0 ETDRS letters, at 12 months. Dashed lined = fitted simple linear regression line. BCVA, best-corrected visual acuity; ETDRS, early treatment diabetic retinopathy study.

The univariate and multivariate analysis results for outcomes at 12 months are summarized in Table 3. Mean final BCVA was 51 ETDRS letters. The final multivariate linear regression model included only better baseline BCVA as a statistically significant (*p* < 0.001) predictor of higher BCVA score (Figure 1(a)). Thirty-four (29.1%) patients ended the first year of treatment with BCVA ≥ 65 ETDRS letters. Once again, the final multivariate model for this outcome included only baseline BCVA (*p* < 0.001), with each additional letter at diagnosis increasing the odds of achieving such a final BCVA score by 1.1-fold (95% CI 1.1, 1.2) (Figure 1(a)).

Mean BCVA variation during the first year was +3.8 ETDRS letters, and seventy (59.8%) patients showed BCVA variation > 0 ETDRS letters. In multivariate analysis, worse baseline BCVA was the only statistically significant predictor of higher BCVA variation (*p* < 0.001) (Figure 1(b)) and of higher probability of visual gain (*p* = 0.001), with each letter increase at baseline changing the odds of gaining vision by 0.96-fold (95% CI 0.94, 0.99) (Figure 1(b)).

### 3.2. Predictors for Outcomes at 60 Months

**Figure 2:**
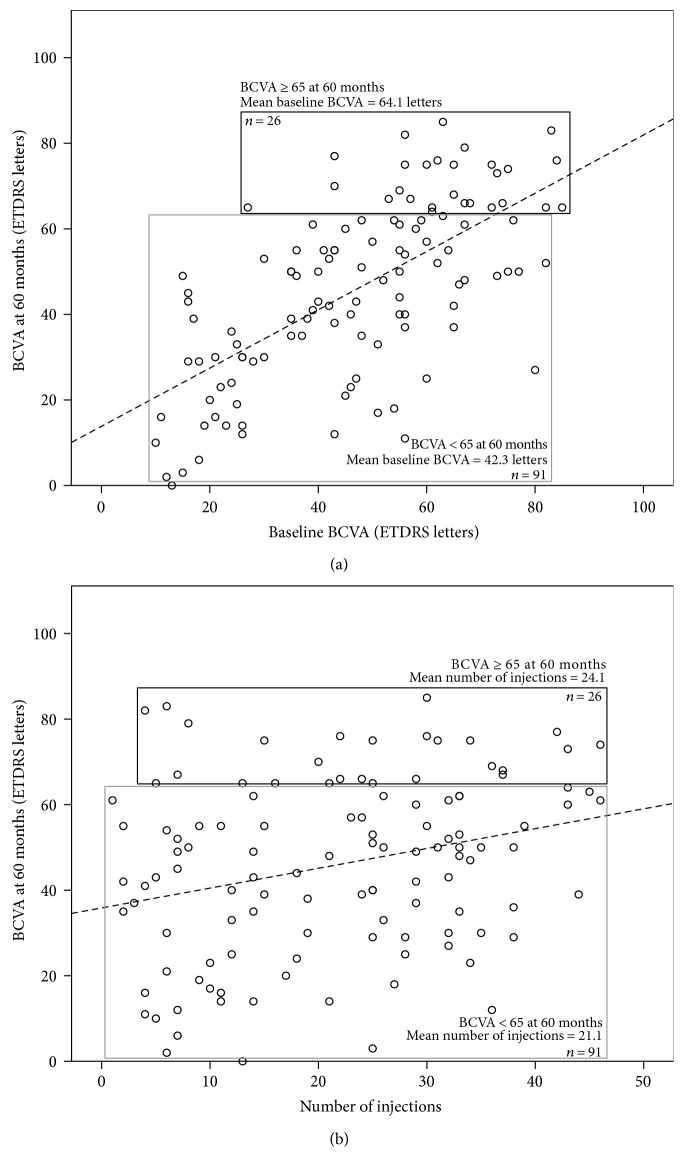
Predictive variables of final best-corrected visual acuity at 60 months. (a) Better BCVA at baseline predicted higher final BCVA score and greater likelihood of achieving 65 ETDRS letters or better, at 60 months. Dashed line = fitted simple linear regression line. (b) Higher number of injections predicted higher final BCVA score, at 60 months. Dashed lined = fitted simple linear regression line. BCVA, best-corrected visual acuity; ETDRS, early treatment diabetic retinopathy study.

The univariate and multivariate analysis results for outcomes at 60 months are summarized in Table 4. Mean final BCVA was 46 ETDRS letters. The final multivariate model for higher final BCVA score included higher baseline BCVA (*p* < 0.001) (Figure 2(a)), no development of subretinal fibrosis during follow-up (*p* = 0.012), and higher number of injections (*p* = 0.014) (Figure 2(b)). Twenty-six (22.2%) patients had BCVA ≥ 65 ETDRS letters at the end of follow-up. The final multivariate model for this outcome included only baseline BCVA (*p* = 0.001), with each additional letter at diagnosis increasing the odds of obtaining that BCVA score by 1.1-fold (95% CI 1.0-1.1) (Figure 2(a)).

**Figure 3:**
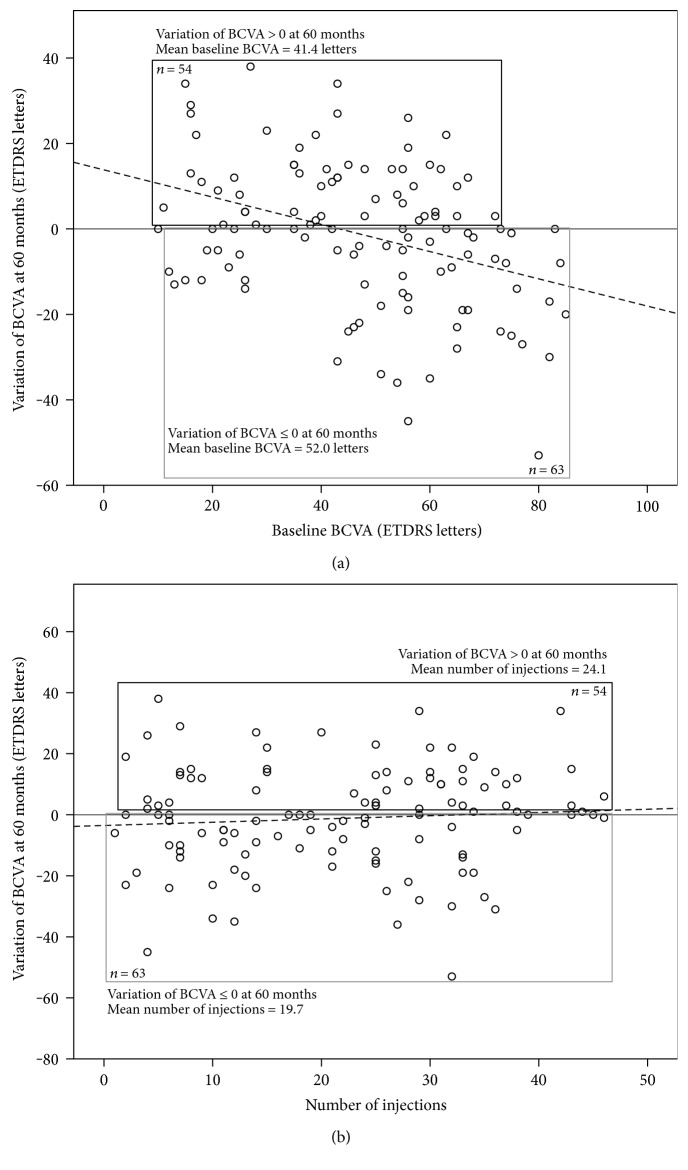
Predictive variables of best-corrected visual acuity variation at 60 months. (a) Worse BCVA at baseline predicted higher BCVA variation and greater likelihood of achieving a variation greater than 0 ETDRS letters, at 60 months. Dashed lined = fitted simple linear regression line. (b) Higher number of injections predicted greater likelihood of achieving a BCVA variation greater than 0 ETDRS letters, at 60 months. Dashed lined = fitted simple linear regression line. BCVA, best-corrected visual acuity; ETDRS, early treatment diabetic retinopathy study.

Mean BCVA variation during the five-year follow-up period was −1.2 ETDRS letters. Once again, in multivariate analysis, the only predictor of higher BCVA variation was worse baseline BCVA (*p* < 0.001). (Figure 3(a)). Finally, fifty-four (46.2%) patients showed BCVA variation > 0 ETDRS letters, and the final multivariate model for this outcome included the following: baseline BCVA (*p* < 0.001) (Figure 3(a)), with each letter increase at diagnosis changing the odds of achieving this endpoint by 0.96-fold (95% CI 0.93–0.98); age at diagnosis (*p* = 0.016), with each additional year changing the odds by 0.92-fold (95% CI 0.87–0.98); gender (*p* = 0.014), with female gender increasing the odds 3.0 times (95% CI 1.3–7.4); and number of injections (*p* = 0.018) (Figure 3(b)), with each additional injection increasing the odds by 1.1-fold (95% CI 1.0-1.1).

## 4. Discussion

Baseline BCVA was a significant and independent predictor of all visual outcomes after one and five years of anti-VEGF therapy. Higher baseline BCVA predicted higher final BCVA and higher likelihood of final BCVA ≥ 65 ETDRS letters, but lower BCVA variation and lower likelihood of gaining vision. These real-world findings confirm what has been shown by the pivotal clinical trials, including the ANCHOR [4] and the MARINA [5] studies, where baseline BCVA was the strongest predictor of visual outcomes, and the CATT study [6] as well. Patients with higher visual acuity at diagnosis have less room for improvement and therefore tend to experience smaller gains with treatment, a phenomenon that has been termed the ceiling effect [4]. However, they are more likely to maintain with treatment a good level of vision that is compatible with daily activities such as reading and driving. This emphasizes the importance of early detection of nAMD, even in this era of highly effective treatment.

Although, at one year of follow-up, no other factor besides baseline BCVA was significant in multivariate analysis, at five years, younger age at diagnosis, female gender, higher number of injections, and no development of subretinal fibrosis were also independently associated with better visual outcomes.

Age at diagnosis was the third strongest predictor of visual outcomes in the ANCHOR [4] and the MARINA [5] trials (after baseline BCVA and CNV lesion size). Its importance was also confirmed by the CATT study [6]. In accordance with these results, we found that younger age independently correlated with a higher likelihood of gaining vision in response to treatment. This does not seem surprising as, in older patients, the retinal structural damage and functional decline related to age may limit the recovery potential.

On the other hand, while, according to the pivotal clinical trials [4, 5], gender has no impact on visual acuity endpoints, our results suggest that female patients are more likely to gain vision with treatment. It is possible that, for some unknown biological reason, women respond better to anti-VEGF agents or have greater potential for recovery, but future studies are needed to confirm or refute this association.

A higher number of injections also was an independent predictor of higher final BCVA and higher likelihood of visual improvement. These findings are in agreement with the CATT study [7], which showed that treatment as needed resulted in less gain in visual acuity compared to monthly injections. However, it should be noted that patients included in our study may have been treated with a PRN regimen, a treat-and-extend regimen or with both sequentially, and the influence of the number of injections on outcomes might vary according to the treatment strategy employed. Nevertheless, it appears that, regardless of the regimen adopted, all efforts should be made to avoid undertreatment, in order to optimize visual outcomes [8].

Finally, even after adjusting for other factors through multivariate analysis, the development of subretinal fibrosis during follow-up predicted worse final BCVA. This finding underlines the need for additional therapeutic agents that target other aspects of nAMD pathogenesis besides angiogenesis, such as fibrosis [1, 9].

The influence of treatment delay on visual outcomes of anti-VEGF therapy deserves consideration. We did find, in univariate analysis, some association between treatment delay and final BCVA score and BCVA variation at five years. However, information regarding the interval between the onset of symptoms and the first anti-VEGF injection was missing in the clinical records of nearly half the patients (49%), and, for this reason, we decided not to include treatment delay in multivariate analysis. It is intuitive to suppose that earlier treatment, initiated before the disease has caused irreversible damage to the photoreceptors and surrounding structures, leads to the best results. However, this is difficult to demonstrate because assessment of treatment delay relies on information provided by the patients, who are often uncertain of when the symptoms appeared; additionally, symptom awareness depends on the visual acuity of the fellow eye. This difficulty is even greater in retrospective studies, because treatment delay is frequently not reported by clinicians in a standardized manner [10], as has been shown by our study. Thus, the interval between onset of symptoms and first anti-VEGF injection is usually not analyzed in clinical trials [10] and is not adequately evaluated by retrospective studies. On the other hand, a prospective, clinic-based study, which evaluated visual outcomes after 6 months of anti-VEGF treatment, demonstrated that longer treatment delay from first symptoms of CNV was, in multivariate analysis, a highly significant predictor of adverse visual outcome, by limiting the ability to gain vision with treatment [10].

In this era of anti-VEGF therapy, the effect of CNV lesion type on treatment response, if any, is still unclear [11]. Photodynamic therapy with verteporfin appeared to work best for predominantly classic CNV, but the beneficial impact of anti-VEGF agents is clear among all lesion types and whether there is any influence of lesion type on treatment response remains to be established. One study showed that, in comparison with occult CNV, predominantly and minimally classic lesions were associated with worse final visual acuity [6], and, in another one, eyes with predominantly classic CNV were the ones that improved more under treatment [12]. Retinal angiomatous proliferation lesions have also been reported to be associated with greater visual gain [6]. In our study, however, CNV lesion type was not an independent predictor of any visual outcome, at one or five years of follow-up.

Other factors analyzed in our study were also not significant in multivariate analysis. For example, the presence of subfoveal hemorrhage at baseline did not independently influence visual outcomes. Interestingly, a cohort study within the CATT [6] reported similar results and, thus, the authors concluded that eyes with hemorrhage may be expected to improve. In addition to this, we did not find the development of RPE tear during follow-up to have any relevant impact on visual prognosis, but it should be noted that the number of patients who developed RPE tear in our study was very small and this may have limited our conclusions.

The strengths and limitations of our study should be considered. We analyzed numerous factors, both at baseline and during follow-up, which could potentially influence response to anti-VEGF therapy. Visual outcomes were evaluated over a long period of time, as all patients included in our study were followed-up for at least five years. On the other hand, the retrospective nature of our study and the reliance of data collection on patients' records, which were not always written in a standardized manner, may have limited our conclusions. Additionally, patients included could have been treated with more than one anti-VEGF agent and with two different treatment regimens.

## 5. Conclusions

In summary, our study showed, in a real-world setting, that baseline BCVA influences final absolute BCVA and BCVA variation at one and five years, while age at diagnosis, gender, number of injections, and development of subretinal fibrosis are independent predictors of visual outcomes after five years of treatment of nAMD with anti-VEGF agents. The identification of predictors of response to anti-VEGF therapy is essential, not to exclude any group of patients from treatment but to adjust expectations for visual outcomes, to optimize treatment, and to further deepen our understanding of nAMD.

## Figures and Tables

**Table 1:** Baseline characteristics of the patients.

Number of patients	117
Women, *n* (%)	68 (58.1)
Mean age at diagnosis ± SD, years	75.70 ± 7.03
Age at diagnosis (range), years	54–89
Mean BCVA ± SD, ETDRS letters	47 ± 19.6
CNV lesion subtype, *n* (%)	
Occult	55 (47.0)
Minimally classic	35 (29.9)
Predominantly classic	16 (13.7)
Retinal angiomatous proliferation	8 (6.8)
Not classified	3 (2.6)
Mean CFT ± SD, *μ*m	311.37 ± 140.85
Subfoveal hemorrhage, *n* (%)	10 (8.5)
Subretinal fibrosis, *n* (%)	9 (7.7)

SD: standard deviation; ETDRS: early treatment diabetic retinopathy study; BCVA: best-corrected visual acuity; CNV: choroidal neovascularization; CFT: central foveal thickness.

**Table 2:** Follow-up characteristics of the patients.

Number of CNV-ME, mean ± SD	37.5 ± 6.8
12 months	9.3 ± 2.0
24 months	8.0 ± 2.0
36 months	7.2 ± 2.1
48 months	6.9 ± 1.9
60 months	6.1 ± 2.2
Missed CNV-ME, mean ± SD	1.6 ± 1.7
Due to disease, *n* (%)	111 (59.0)
Due to unknown reasons, *n* (%)	77 (41.0)
Patients with at least 1, *n* (%)	78 (66.7)
Patients with more than 1, *n* (%)	49 (41.9)
Number of injections, mean ± SD	21.7 ± 12.4
12 months	6.0 (2.4)
24 months	4.2 (3.1)
36 months	3.7 (3.1)
48 months	4.0 (3.7)
60 months	3.8 (3.7)
Missed injections, *n*	27
Patients with at least 1, *n* (%)	25 (21.4)
Patients with more than 1, *n* (%)	2 (1.7)
Treatment suspensions, mean ± SD	5.3 ± 3.0
Due to medical indication, *n* (%)	562 (90.8)
Without medical indication, *n* (%)	57 (9.2)
Treatment reinitiations, mean ± SD	4.7 ± 3.1
Patients needing further treatment at the end of follow-up, *n* (%)	51 (43.6)
Development of atrophy, *n* (%)	83 (70.9)
During treatment, *n* (%)	52 (62.7)
During suspension with medical indication, *n* (%)	31 (37.3)
Development of RPE tear, *n* (%)	9 (7.7)
During treatment, *n* (%)	8 (88.9)
During suspension with medical indication, *n* (%)	1 (11.1)
Development of subretinal fibrosis, *n* (%)^a^	75 (68.5)
During treatment, *n* (%)	61 (81.3)
During suspension with medical indication, *n* (%)	14 (18.7)
Sudden massive hemorrhages	
Episodes during treatment, *n* (%)	24 (51.1)
Episodes during suspension with medical indication, *n* (%)	23 (48.9)
Patients with at least 1 episode, *n* (%)	38 (32.5)
Patients with more than 1 episode, *n* (%)	9 (7.7)

^a^ *n* = 108 patients, as 9 people had subretinal fibrosis at baseline. CNV-ME: choroidal neovascularization-medical examinations; RPE: retinal pigment epithelium.

**Table 3:** Univariate and multivariate analysis for prediction of visual outcomes at 12 months.

Variables at baseline	BCVA score		ΔBCVA score		BCVA score ≥ 65		ΔBCVA score > 0	
	Univariate analysis (*p* value)	Multivariable analysis (*p* value)^b^	Univariate analysis (*p* value)	Multivariable analysis (*p* value)^b^	Univariate analysis (*p* value)	Multivariable analysis (*p* value)^e^	Univariate analysis (*p* value)	Multivariable analysis (*p* value)^e^
Gender	0.439^a^	—	0.508^a^	—	0.921^d^	—	0.615^d^	—
Age	0.087^b^	0.083	0.177^b^	0.084	0.526^e^	—	0.094^e^	0.090
Treatment delay	0.909^b^	—	0.634^b^	—	0.989^e^	—	0.482^e^	—
Baseline BCVA	**<0.001** ^c^	**<0.001**	**0.001** ^c^	**<0.001**	**<0.001** ^f^	**<0.001**	**0.001** ^f^	**0.001**
CNV lesion subtype	**0.041** ^b^	—	0.080^b^	—	0.121^g^	—	0.277^g^	—
CFT	**0.003** ^b^	0.538	0.927^b^	—	**0.047** ^e^	0.645	0.576^f^ ^‘^	—
Subfoveal hemorrhage	0.538^a^	—	0.340^a^	—	1.000^g^	—	1.000^g^	—
Subretinal fibrosis	0.603^a^	—	0.743^a^	—	1.000^g^	—	0.481	—

BCVA: best-corrected visual acuity; Δ: variation; CNV: choroidal neovascularization; CFT: central foveal thickness; *p* value was from the following: ^a^independent-samples *t*-test; ^b^one-way ANOVA; ^c^Kruskal-Wallis test; ^d^Pearson chi-square; ^e^binomial logistic regression; ^f^linear-by-linear association; ^g^Fisher's exact test; variables with *p* < 0.2 in univariate analysis were included in the multivariable analysis; variables with *p* < 0.05 in multivariable analysis were included in the final multivariable model for predictive factors of designated outcome.

**Table 4:** Univariate and multivariate analysis for prediction of visual outcomes at 60 months.

Variables at baseline	BCVA score		ΔBCVA score		BCVA score ≥ 65		ΔBCVA score> 0	
	Univariate analysis (*p* value)	Multivariable analysis (*p* value)	Univariate analysis (*p* value)	Multivariable analysis (*p* value)	Univariate analysis (*p* value)	Multivariable analysis (*p* value)	Univariate analysis (*p* value)	Multivariable analysis (*p* value)
Gender	0.842^a^	—	**0.039** ^a^	0.246	0.161^d^	0.251	**0.013** ^d^	**0.014**
Age	**0.047** ^b^	0.617	0.109^b^	0.200	0.770^e^	—	0.088^e^	**0.016**
Treatment delay	0.151^b^	—	0.009^b^	—	0.839^e^	—	0.795^e^	—
Baseline BCVA	**<0.001** ^b^	**<0.001**	**<0.001** ^b^	**<0.001**	**<0.001** ^f^	**0.001**	**0.004** ^f^	**<0.001**
CNV lesion subtype	**0.002** ^b^	—	0.801^b^	—	0.078^g^	—	0.990^g^	—
CFT	**0.003** ^b^	0.218	0.907^b^	—	0.148^f^	0.781	0.702^f^	—
Subfoveal hemorrhage	0.957^b^	—	0.920^b^	—	1.000^g^	—	0.748^g^	—
Subretinal fibrosis	0.820^c^	—	0.212^a^	—	0.681^g^	—	0.731^g^	—
*Variables during follow-up*								
Number of CNV-ME	**0.017** ^b^	0.533	0.302^b^	—	**0.014** ^f^	0.084	0.259^f^	—
Number of missed CNV-ME	0.822^b^	—	0.901^b^	—	0.919^f^	—	0.980^f^	—
Number of injections	**0.002** ^b^	**0.014**	0.401^b^	—	0.263^f^	—	0.060^f^	**0.018**
Number of missed injections	0.064^b^	0.253	0.676^b^	—	0.630^f^	—	0.160^f^	0.233
Number of treatment suspensions	0.078^b^	0.960	0.957^b^	—	0.070^f^	0.954	0.400^f^	—
Development of atrophy	0.113^c^	0.745	0.326^c^	—	0.625^c^	—	0.221^f^	—
Development of RPE tear	0.120^c^	0.226	0.830^c^	—	0.204^g^	—	0.731^g^	—
Development of subretinal fibrosis	**<0.001** ^c^	**0.012**	0.224^a^	—	0.052^d^	0.123	0.512^f^	—
Number of sudden massive hemorrhages	**<0.001** ^b^	0.075	0.289^b^	—	**0.017** ^f^	0.306	0.278^f^	—

BCVA: best-corrected visual acuity; Δ: variation; CNV: choroidal neovascularization; CFT: central foveal thickness; CNV-ME: choroidal neovascularization medical examination; RPE: retinal pigment epithelium; *p* value was from the following: ^a^independent samples Mann–Whitney *U* test; ^b^one-way ANOVA; ^c^independent-sample *t*-test. ^d^Pearson chi-square; ^e^linear-by-linear association; ^f^binomial logistic regression; ^g^Fisher's exact test; variables with *p* < 0.2 in univariate analysis were included in the multivariable analysis; variables with *p* < 0.05 in multivariable analysis were included in the final multivariable model for predictive factors of designated outcome.
